# A Patient with Complex Gout with an Autoinflammatory Syndrome and a Sternoclavicular Joint Arthritis as Presenting Symptoms

**DOI:** 10.1155/2020/5026490

**Published:** 2020-01-31

**Authors:** M. M. Fedeli, M. Vecchi, P. Rodoni Cassis

**Affiliations:** ^1^Department of Internal Medicine, Ospedale La Carità, Locarno, Switzerland; ^2^Centro Medico, Chiasso, Switzerland; ^3^Department of Radiology, Clinica Sant'Anna, Lugano-Sorengo, Switzerland; ^4^Department of Radiology, Clinica Ars Medica, Gravesano, Switzerland

## Abstract

A 50-year-old man presented to the emergency department with widespread pain, especially at the chest level, fever, and night sweats. Physical examinations revealed a swelling with localized pain in the left sternoclavicular joint. Laboratory tests showed a CPR of 134 mg/l and an ESR of 70 mm/h. The patient's anamnesis is, for a chronic gouty arthritis, poorly controlled type 2 diabetes and a lumbosacral radicular syndrome. Home therapy includes metformin, sitagliptin, gliclazide, naproxen with partial benefit on pain, and febuxostat. Differential diagnoses of sternoclavicular swelling include infection, crystal or psoriatic arthropathy, tumor pathology, SAPHO syndrome, and osteoarthritis. An ultrasound scan performed at the thoracic level showed the presence of effusion in the sternoclavicular joint. A thoracoabdominal CT scan, performed in doubt of neoplasias, shows no masses but osteostructural nonspecific alterations of the sternoclavicular joint. We performed a dual energy CT (DECT) which reports a gouty arthropathy at the sternoclavicular joints (in the literature, only three similar cases are proved). Because of the poor therapeutic effects using febuxostat and systemic corticosteroids, the patient was treated with anakinra, an interleukin 1 receptor antagonist, which led, 6 months after the event, to a total remission.

## 1. Case Report

R. is a 50-year-old patient who has, since a month, a state of general malaise characterized by widespread pain and intermittent episodes of fever and night sweats. He refers fever with stakes up to 39°C in two days prior to hospitalization at the Department of Rheumatology. The pains are especially at night to the chest level. Despite a home drug therapy with NSAID (nonsteroidal anti-inflammatory drug), symptoms do not regress.

The patient's anamnesis is for a chronic gouty arthritis characterized by the presence of tophi and frequent arthritis; the first gouty attack was in 2011 on the ankles treated with steroids. In 2014, he has undergone surgical operation with the removal of tophaceus masses [1] at the sixth compartment of the right hand because of severe pain and losing range of motion in the affected joint.

**Figure 1:**
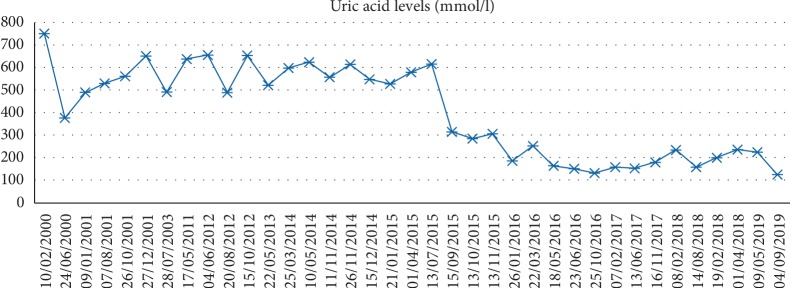
Monitoring uric acid levels over the years.

He also had high uric acid levels (Figure 1) as he consumed febuxostat (allergic to allopurinol).

He is also known for type 2 diabetes (diagnosis of 2011) in pharmacological treatment with metformin, sitagliptin, and gliclazide poorly controlled because of wrong eating habits without a regular physical activity. In fact, in physiological anamnesis, the patient declares he drinks 2–3 liters of carbonated soft drinks a day.

**Figure 2:**
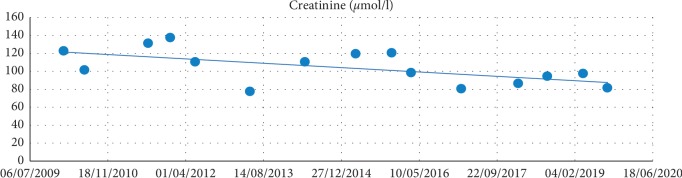
Monitoring renal function levels over the years.

He has a class 1 obesity (BMI 31.5 Kg/m^2^) that is why he underwent gastric banding surgery in 2004 with mediocre success. Another cardiovascular risk factor is a chronic renal failure (Figure 2) G3a (KDIGO, 2012) [2] due to diabetic nephropathy (albuminuria grade A2) [3].

Lastly, he suffered from lumbosacral radicular syndrome, irritative L3 on the left side.

On examination, the patient is in good general condition, 102 kg weight, 180 cm height, with eupnea, normal heart rate, normal heart rhythm, and normal cardiac rhythm. Clinically, there was a florid inflammation at the level of the first metatarsophalangeal joint of the left foot with a swelling of the left ankle without pain on pressure or mobilization. The knees appear bilaterally swollen, warm to thermotouch, aching with the presence of a joint effusion mostly expressed on the left. We deduce inflammation to the metacarpophalangeal joints (II, III, and IV) of the left hand where, in the past, tophi had been removed surgically. The movement in extension of the left elbow is deficient. Lumbar spine mobility is reduced by 1/3 in the lateral-bilateral flexion and in forward flexion with soreness at the lumbosacral level. At chest level, there is a swelling with localized pain in the left sternoclavicular joint. Valid heart sounds, no murmurs were observed. At lung auscultation, there was vesicular murmur spreading ubiquitously. The abdomen is treatable and painless on palpation. No evidence of associated neurological deficits.

The electrocardiogram revealed no significant alterations. Laboratory tests show a CRP of 134 mg/l and an ESR of 70 mm/h. Uric acid is within the normal limit (263 mmol/l).

The differential diagnosis of sternoclavicular swelling:

- Infectious arthropathy
- Crystals arthropathy (uric acid or calcium pyrophosphate crystals)
- Tumor pathology
- Psoriatic arthropathy
- SAPHO syndrome (synovitis, acne, pustulosis, hyperostosis, and osteitis)
- Osteoarthritis

Proceeding by elimination, we have eradicated from our list psoriatic arthritis in a patient who showed no skin changes, and with silent family history for this disease. A thoracoabdominal CT scan, performed in doubt of neoplasias, shows no masses but osteostructural nonspecific alterations of the sternoclavicular joint. Procalcitonin is negative reducing the chance of bacterial infection. Trauma was not reported in the recent period. We have also excluded osteoarthritis because of the absence of the classical radiological signs (narrowing of joint space, osteophytes, subchondral bony sclerosis, and subchondral cysts) [4, 5].

**Figure 3:**
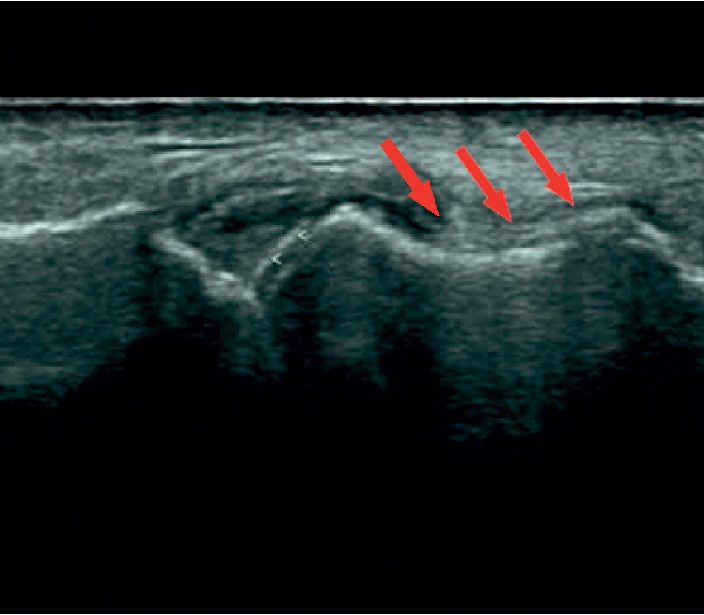
Functional ultrasound scan of the knee.

Therefore, we continued with a functional ultrasound scan to the knee that showed synovitis with joint effusion and the characteristic double contour that means the presence of deposits of urate at the femoral condyles cartilage level (Figure 3) [1].

The synovial fluid, drawn at the knee level, confirmed this hypothesis given the presence of uric acid crystals.

A chest ultrasound showed the presence of effusion in the sternoclavicular joint and thickening of the synovial capsule.

**Figure 4:**
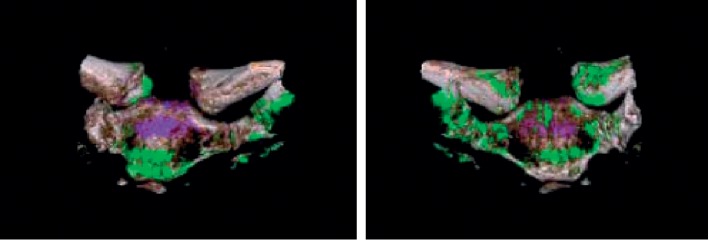
Sternoclavicular details with Dual Energy CT 3D reconstruction.

**Figure 5:**
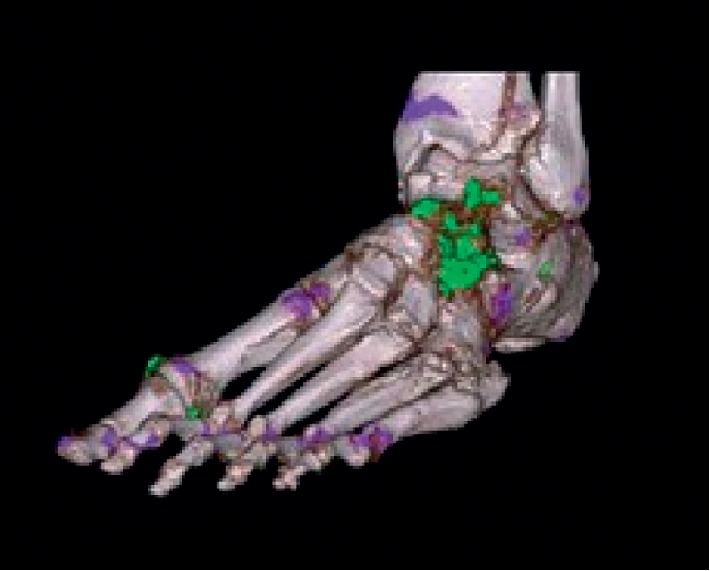
Left foot with Dual energy CT 3D-reconstruction.

Because of the lack of joint fluid, we could not carry out arthrocentesis of sternoclavicular joint. We performed a CT with the DUAL ENERGY (DECT) method, which confirmed the suspicion of gout-originated arthropathy at the sternoclavicular joints, explaining the patient's chest symptoms. Specifically, we show the 3D reconstruction of the dual energy CT of the sternoclavicular joints and the foot (Figures 4 and 5) [1]. The uric acid deposits appear in green, while the bone calcium appears in purple.

**Figure 6:**
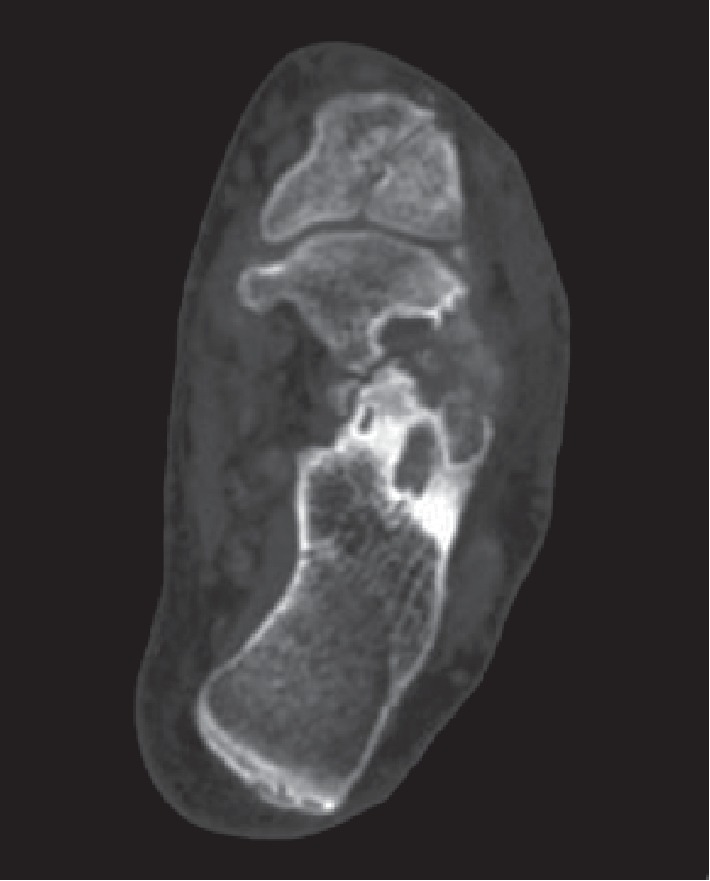
Erosion and intra-articular swelling with tophaceous masses (CT of the left foot).

Our patient has a systemic disease with destructive deforming arthritis. Indeed, it has some tophaceous lesions (Figure 6) [1].

## 2. Discussion

A gouty arthritis with a sternoclavicular localization is a very rare finding (in the literature, only three similar cases are proved [6]). Classically, imagining a classic case of gout, we thought more frequently to the metatarsophalangeal joint (podagra), to proximal and distal interphalangeal, to elbow and knee [1, 5, 7] Our patient had an intermediate probability of having gout (7.5 points) [8]. In this case, the gold standard for the diagnosis of gout remains arthrocentesis with the detection of uric acid crystals [8]. The diagnostic alternatives include ultrasound, with the classic double contour of the cartilage surface typical of arthropathies with crystal deposits, and the Dual Energy CT [1, 7, 9]. With this method [9], the images are acquired simultaneously, using two different energy levels. Comparing the specific attenuation at 80 and 140 kVp, it is possible to differentiate the chemical composition of tissues subjected to CT. Uric acid crystals are thus differentiated from the bone or from calcium-based dystrophic calcification. Recent studies have shown that CT Dual Energy a sensitivity of 75 to 90% and a specificity between 83 and 93%, respectively [9]. This type of examination, therefore, represents an excellent alternative for the diagnosis of gout and in particular, in the case of clinical doubts or inconclusive microscopic analysis.

The abundant consumption of drinks, given the high-fructose content, may have helped to maintain the hyperuricemia and played a role in causing acute attacks through urea fluctuations [10, 11].

Another useful starting point in clinical practice is the uric acid levels were normal; this indicates how important are the uric acid fluctuations in blood rather than the peak in parallel with acute attacks of gout [7].

At last, a brief mention of therapy. Regarding the acute attack, you should first use AINS or colchicine and, in case of monoarticular attack, also corticosteroid infiltrations. Because of the failure benefit of AINS, the failure tolerance of colchicine and polyarticular localization, we have resorted to prednisone starting with a dosage of 20 mg to scale gradually down. Febuxostat treatment has been kept, and because of the poor therapeutic effects, we decide to use anakinra, an inhibitor of interleukin 1, which led to total remission. The total duration of the gout flare was 6 months.

## 3. Take Home Message

Summarizing, the key points are:

- Gout can occur in a generalized and systematic way and affect any joint [1]
- Dual-Energy CT or ultrasound scan are the best functional tests chosen in cases of doubt or for special localizations of gout [5, 7]
- The abuse of carbonated soft drinks, rich in fructose, is a risk factor comparable with beer for gout [10, 11]
- Normal uric acid levels do not exclude a gouty attack [5, 7]
